# Online Arabic Beverage Frequency Questionnaire (ABFQ): evaluation of validity and reliability

**DOI:** 10.1186/s12937-022-00830-9

**Published:** 2023-03-22

**Authors:** Tahrir M. Aldhirgham, Lulu A. Almutairi, Atheer S. Alraqea, Amani S. Alqahtani

**Affiliations:** Executive Department of Research and Studies, Saudi Food and Drug Authority (SFDA), Northern Ring Branch Rd, Hitteen Dist, Riyadh, 7148-13513 Saudi Arabia

**Keywords:** Online questionnaire, Validity, Reliability, Fluid, Beverage, Arabic, MENA-region

## Abstract

**Background:**

Obesity and chronic diseases are significant public health issues in the Middle East and North Africa region. A robust body of evidence demonstrated the association between beverage consumption, obesity, and chronic diseases. Therefore, the assessment of beverage consumption is gaining more interest in health policy development, food industry partnerships, research expansion and community involvement. Although beverage-consumption assessment tools have been developed for various populations, none were developed for the Arabic population. In this study, we developed and validated an online Arabic Beverage Frequency Questionnaire (ABFQ) to assess the total beverage intake among Arabic speaking population.

**Methods:**

A cross-sectional validation study was conducted among healthy adults aged between 18 and 55 years. Participants (*n* = 49) completed a 24-item ABFQ on two occasions and provided one 24-h urine sample. For validity, total beverage consumption (ABFQ1) was assessed against a 24-h urine sample using an osmolality test and correlation analysis. Reliability was assessed by comparing the participants’ consumption in total and for every 24 individual items from ABFQ1 with the total and individual items in ABFQ2 using correlation and paired sample t-test.

**Results:**

The average daily consumption of beverages was 1504 ml/day, while the average urine osmolality/kg was 614. The validity assessment between ABFQ and urine osmolality indicates a negative correlation. However, the correlation was week and not statistically significant (r_s_ = -0.2, *p* = 0.12). In reliability test, correlation analysis was positive and acceptable in all beverage categories (r_s_ = 0.4 − 0.9; all *p* < 0.05) except flavored milk (r_s_ = 0.2; *p* < 0.181) and sweetened coffee (r_s_ = 0.3; *p* < 0.022). Furthermore, no significant differences were found between the means of total consumption in both ABFQ1 and ABFQ2.

**Conclusions:**

The finding of this study suggest that the ABFQ is a reliable reproducible tool for assessing beverage consumption among Arabic-speaking consumers. However, the survey could not be validated using 24-h urine osmolality only and other methods such as multi dietary records may use in future re-assessment.

## Introduction

Globally, non-communicable diseases (NCDs), including cardiovascular diseases, cancers, chronic respiratory diseases and diabetes, cause 41 million deaths, corresponding to 71% of all deaths annually [1]. In the Middle East and North African countries (MENA), the prevalence and mortality related to NCDs are similar to the global situation [2]. In addition, the region has six of the ten countries with the highest diabetes prevalence in the world [3]. Furthermore, obesity prevalence in most Arab countries grew to 55% and 30% in adult females and males, respectively [4].

NCDs have many metabolic risk factors, including obesity, hyperglycemia and hyperlipidemia that can be developed due to behavioral risk factors such as physical inactivity and unhealthy diet and beverage consumption. A considerable body of evidence demonstrated the association between beverage consumption and NCDs. For instance, high consumption of Sugar-sweetened beverages was found to be associated with obesity and diabetes mellitus in adults and children [5–12], hypertension, and coronary heart diseases [13–16]. Nevertheless, beverage intake is a significant contributor to health and well-being. For instant, water is essential for the human body’s biological functions, general health, and prevention of chronic disease [17, 18]. Moreover, caffeinated beverages have intake-based health risks and benefits, especially in patients with cardiovascular diseases and hypertension [19, 20].

Given the importance of beverages as part of daily diet and their contribution to health and disease, an accurate assessment of beverage intake is required. Beverage consumption had been assessed previously using nutritional assessment tools, such as food diary recalls and food intake records (FIR) [21, 22]. However, these methods are designed to assess food intake in general and might be imprecise for beverage consumption assessment [23–25]. Accordingly, various beverage consumption frequency-based assessment tools have been developed to assess beverage consumption in languages other than Arabic [24–28]. The developed tools were paper-based surveys that relied on participants estimation of portion size. Recently, one online tool was developed to assess the Canadian population’s beverage consumption in the English language [29].

The Middle East and North Africa (MENA) region includes 21 countries with a total population of approximately 464 million [30]. Arabic is the main language, and Islam is the main religion in MENA countries. Additionally, they share similar social, political, economic, cultural and heritage [31]. Accordingly, some food and beverage consumption is prohibited, such as alcohol [32, 33]. Industrial food processing and international trade marketing also affected the dietary patterns, and all different types of food and beverage are available in the markets. However, the populations in these countries maintained many traditional beverages such as Laban (fermented milk), Cawah ( different types of traditional Coffee drink), and zahorate ( drink made of different types of herbs and/or plant leaves). Local traditional beverages differ among Arab countries in their consumption patterns, recipes, preparation methods and availability [31].

To this end, there are no reliable measures of beverage consumption validated in Arabic and specifically for use within the Arabic-speaking MENA region population. Therefore, this study aimed to develop and validate the first online Arabic Beverage Frequency Questionnaire (ABFQ) to assess the total beverage intake among Arabic speaking population.

## Materials and methods

### Study design

**Fig. 1:**
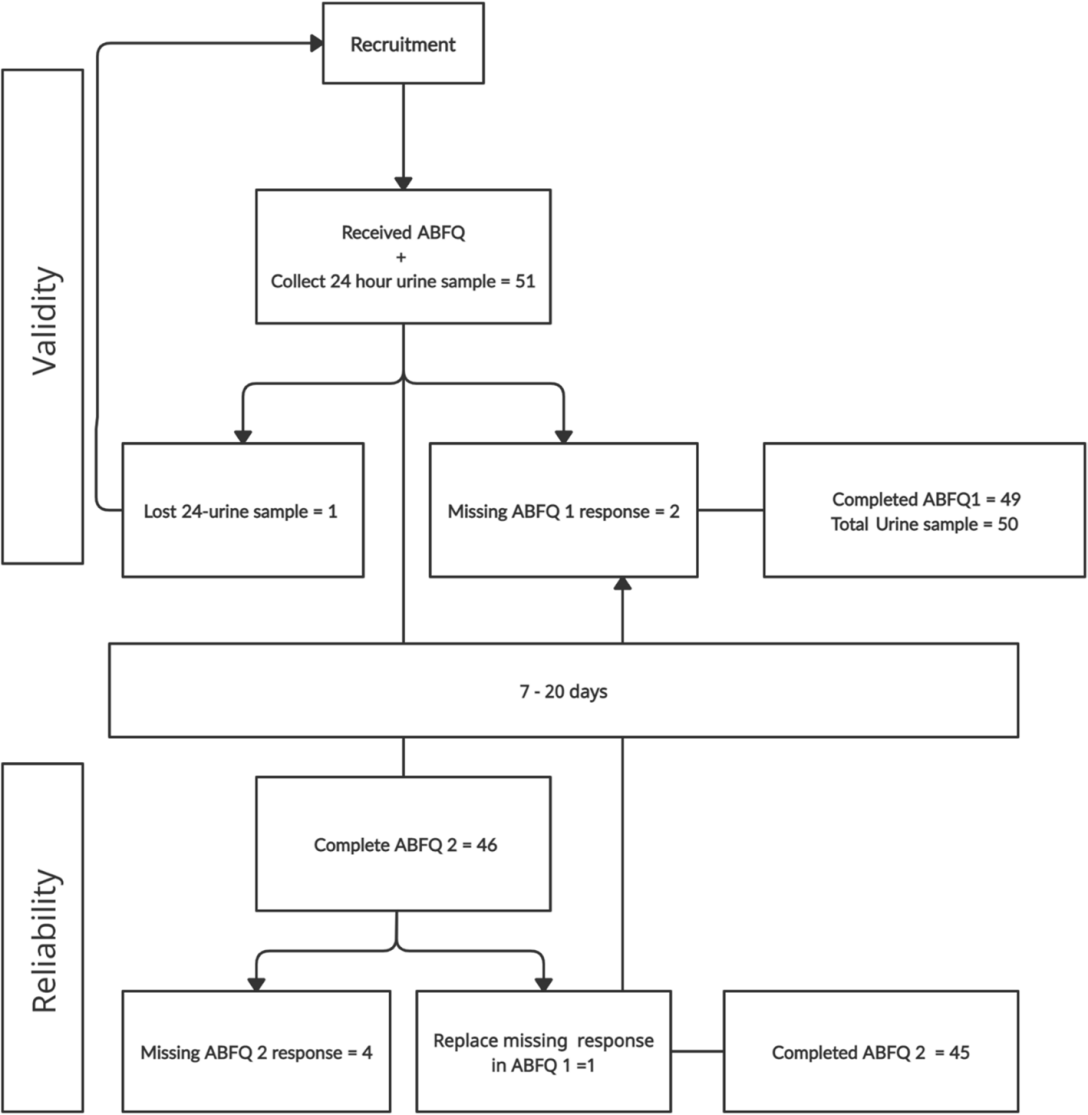
Study design, participants and recruitment process

A cross-sectional study to test the validity and reliability of a new developed ABFQ was conducted between January 30^th^ and March 23^rd^, 2021, in Riyadh, Saudi Arabia. The study consisted of two parts, 1) a self-administered questionnaire that was filled up by the participants two times in different periods (ABFQ1, ABFQ2), and 2) a lab test of a 24-h urine sample collected from participants. The correlation between the average daily intake value (based on ABFQ1) and urine osmolality value on the same day of ABFQ1 (based on a 24-h urine sample) was evaluated for validity assessment similarly to Ferreira-Pêgo *et.al.* (2016) [24]. While reliability assessment was conducted by comparing the average daily consumption from ABFQ1 with the average daily consumption from ABFQ2. Figure 1. Provide more information on study design, participants, and the recruitment process.

### Participants

A convenient sample of healthy adults (*n* = 51) aged between 18 and 55 was recruited from the local community of Saudi Food and Drug Authority (SFDA), i.e., the employee and their families because they may be among the best community group will follow the study instructions. Participants who were over 18 years up to 55 years were Arabic speaking and healthy (no history of chronic diseases such as heart disease, hypertension, diabetes, kidney diseases or health conditions that may influence the fluid intake or output either because of the condition, its treatment or complication) were included in the study. The study protocol was approved by the SFDA Institutional Review Board.

### Recruitment process

Targeted participants were invited verbally to the study, and the study protocol was explained in detail by one of the trained research teams. Information provided about the method of communication, the need for filling two ABFQ versions, the duration between questionnaires, the need for urine sample collection, and the duration and instruction of urine sample collection. After Enrollment, participants were provided with a consent form, along with a urine sample container. On the next weekend (Saturday), the participants received the questionnaire through the short message service (SMS) containing the first ABFQ survey (ABFQ1) and were asked to collect the urine sample on the same day. The Urine sample collection determined on a weekend day (Saturday) to avoid any inconvenience during urine sample collection at work or university and to ensure completeness of the sample (Weekend days in Saudi Arabia is Friday and Saturday; most activities done on Friday). After 7 days, the participants received another link of ABFQ2 (second survey). A three-reminder message was sent to the participants who did not complete ABFQ2 before being withdrawn from the study.

### Measurements

#### Urine samples collection

The aim of using biological measures is to provide objective assessment and to help in avoiding the bias that may result from self-reporting [23, 25]. To the best of our knowledge, no golden standard biomarker reflects the change in hydration status according to changes in dietary fluids intake [34]. However, urine osmolality in a 24-h urine sample is considered the most suitable body hydration status biomarker for individuals because it represents the net sum of water gains, losses, and neuroendocrine regulatory responses [35]. To measure the urine osmolality in a 24-h urine sample, sample containers (2000 ml) handled to participants who were expressed interest in participating and provided verbal consent. Then, participants received verbal instructions on how to collect 24-h urine samples. Each participant was asked to collect the sample on the first next morning after discarding the first urine sample, then collect all-after samples for the next 24 h, including the first urine sample of the next day. Afterwards, participants dropped the sample containers at the previously determined collecting point. All samples were transferred to an approved independent laboratory with the capacity to deal with urine samples and the necessary facilities to run the 24-h urine osmolality test (Cryoscopic Osmometer). Targeting healthy adults (free of chronic diseases or health condition that may influence fluid intake or output) ensured that osmolality test will not be affected by kidney disfunction or other physiological condition. Also, ensured avoiding the use of medications and its possible effect on fluid output or urine osmolality. The study conducted at the middle of winter season (in Saudi Arabia, from December to April), therefore, questionnaire and urine sample test conducted in similar weather conditions and similar level of fluid consumption.

The device used is OSMO STATION, OM-6060, an automatic osmometer that measures the osmolality in different samples, including blood and urine. The determination method is freezing point depression osmometry based on the principle of lowering the solution freezing point caused by the solute. A sample of the specimen (200 μl) to be analyzed is aspirated into the sample tube, which is then placed in the cooling chamber of the osmometer. The sample is super cooled below the freezing point. Then crystallization is initiated by rapidly vibrating the sample to seed it with air bubbles. After seeding, the sample temperature rises because of the heat of fusion released during the freezing process. The temperature rises until the equilibrium plateau is reached. During the equilibrium plateau, only a small fraction of the water is frozen. The sample continues to freeze as the temperature begins to decrease again because of the colder environment until it provides the osmolality value. Each sample container was labelled with a unique number entered by participants at the beginning of both questionnaires (ABFQ1, ABFQ2) to connect participants samples with both questionnaires.

#### Arabic Beverage Frequency Questionnaire (ABFQ)

The ABFQ is an online-based tool developed to assess the total beverage intake among Arabic-speaking consumers. The included beverage categories were as follows; water, fresh fruit juice, fresh vegetable juice, caned 100% juice, sweetened juice, milk juice, low-fat milk, free fat milk, flavored milk, soft drinks, Iced tea, artificially sweetened soft drinks, artificially sweetened iced tea, unsweetened tea, sugar milk tea, unsweetened coffee, sweetened coffee, unsweetened black coffee, sweetened black coffee, flavored coffee, malt drinks, artificially sweetened energy drinks, sweetened energy drinks, sports drinks. In addition to two open-ended question sets to declare any other beverage that was not included in the questionnaire. Sweetened beverage meant beverage sweetened with sugar, while artificially sweetened beverage meant beverage sweetened with non-nutritive sweeteners.

The beverage consumption assessment questions were adapted from previous studies [24, 25, 36]. However, the categories were adjusted based on energy and macronutrient content (calories and sugar content) using Food Data Central of USDA data [37], nutrition fact labels, and the calories content in the coffee shops and fresh juice shops menus.

**Fig. 2:**
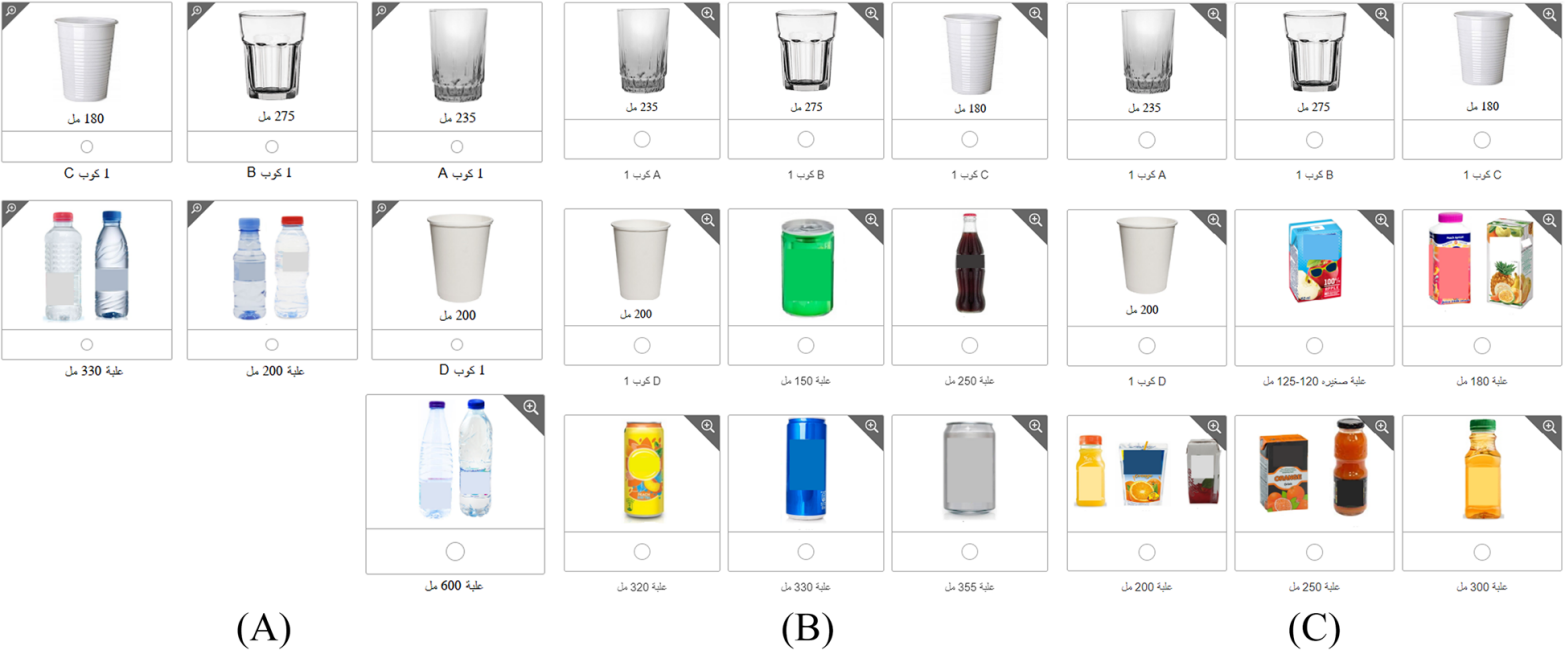
Example of portion size pictures used in ABFQ, **A** different portion size for water, **B** different portion size for soft drinks, **C** different portion size for juices

For each category, the consumption frequency was quantitatively assessed by asking, “during the last 30 days, how many times you drink…”. The response was categorized into (never, once a month, twice a month, three times a month, Once a week, 2–3 times a week, 4–6 times a week, once a day, twice a day, 3 times a day. Then, the amounts consumed were assessed by asking, “how much did you usually drink each time?” in combination with a series of pictures of the common serving size of each type of beverage, such as common cups, bottles, and containers with the volume in (ml) below the picture (see Fig. 2). Pictures adapted from product company websites or were taken for the study purpose. Option “More” was available in both questions with an open text box. During pilot testing of the ABFQ, the average administration time was 10 min.

To score the ABFQ, frequency (“How often”) is transformed to the unit of times per day, then multiplied by the amount consumed (“How much each time”) to find the beverage’ average daily intake in (ml) using the following formula: [(consumption frequency per month / 30 days) x amount consumed each time (ml) = average daily intake (ml/day)]. Total energy and sugar content of beverages could not be determined because of the lack of a local database of beverage nutrient content or food composition tables.

#### Sociodemographic characteristics

To assess other covariates, participants were asked about sociodemographic statuses, including age, gender, education, marital status, and chronic disease status and illness history. Furthermore, they were asked if they have any health condition that may influence the fluid intake or output because of the condition, its treatment or complication during the study period. Weight and height were also self-reported, which were used to calculate body mass index (BMI). BMI were categorized into 4 groups [underweight (< 18.5 kg/m^2^), normal weight (18.5–24.9 kg/m^2^), overweight (25.0–29.9 kg/m^2^) and obese (> 30.0 kg/m^2^)] according to World Health Organization (WHO) [38].

### Data analysis

Descriptive statistics (mean ± standard error of the mean; frequencies) were generated for demographic characteristics, average daily intake of total beverage and average daily intake of each beverage category (ml). To assess the validity, the average daily intake of total beverage (ml/day) was investigated by the ABFQ1 compared to urine osmolality in 24-h urine samples using correlational analyses (Spearman’s correlation). Moreover, to examine test–retest reliability, the ABFQ1 responses were compared with ABFQ2 responses using the same correlational analyses (Spearman’s correlation) and paired sample t-test. The significance in all statistical tests was set at *p* < 0.05. Analysis conducted using Stata version 16 (StataCorp. 2019. Stata Statistical Software: Release 16. College Station, TX: StataCorp LLC).

## Result

A total of 49 participants in ABFQ1 and 45 in ABFQ2 were included in the present analysis. At baseline, the mean age of participants was 32 ± 8 years (range 18 to 54 years). The body mass index was widely distributed (mean = 25.6 ± 4.3 kg/m2; range 16.9—35.5 kg/m2), while participants were primarily of “normal” BMI status (55.1%). Of 49 participants, the most reported having a bachelor’s degree (53%) and being unmarried (65.3%). The duration between questionnaires responses ranges between 7 to 17 days (Median: 7 days). More general characteristics of the study participants are summarized in Table 1.

**Table 1:** General characteristics of the study participants

Variables	Frequency (n)	Percentage (%)
**Questionnaire response**		
ABFQ1	49	96
ABFQ2	45	88.2
**BMI**		
Under weight	1	2
Normal weight	27	55.1
Overweight	9	18.3
Obese	12	24.4
**Age groups (years)**		
18 to 25	9	18.3
26 to 35	25	51
36 to 45	11	22.4
46 to 55	4	8.1
**Gender**		
Male	35	71.4
Female	14	28.5
**Education level**		
High School or less	8	16.3
Bachelor’s degree	26	53
Postgraduate	15	30.6
**Marital status**		
Married	16	32.6
Unmarried	32	65.3
Separated or widowed	1	2

BMI were categorized according to World Health Organization (WHO) into 4 groups [underweight (< 18.5 kg/m^2^), normal weight (18.5–24.9 kg/m^2^), overweight (25.0–29.9 kg/m^2^) and obese (> 30.0 kg/m^2^)] [38]

### Relative validity of the questionnaire (ABFQ1 vs urine osmolality test)

The average daily consumption based on the ABFQ was 1504 ml/day, while the average urine osmolality/kg was 614 mOsm/kg. Correlational analysis between total fluid intake (ml/day) based on the ABFQ1 and 24-h urine osmolality (mOsm/kg) yields a negative correlation (r_s_ = -0.2, *p* = 0.12) as would be expected for a possible biomarker of total fluid intake. However, the correlation were week and non-significant. See Table 2.

**Table 2:** Validity assessment (Spearman’s correlation)

	**Mean (min–max)**	**SD**	**Median**	***r***_***s***_	***P* value**
**ABFQ1** (ml/day)	1504 (463–4198)	769	1383	-0.2	0.12
**Urine osmol** (mOsm/kg)	614 (200–1068)	234.1	589		

### Test–retest reliability of the questionnaire (ABFQ1 vs ABFQ2)

Correlation analysis for the questionnaire reliability was positive and acceptable in all beverage categories (r_s_ = 0.4 − 0.9; all p ≤ 0.05) except flavored milk (r_s_ = 0.2; *p* = 0.181) and sweetened coffee (r_s_ = 0.3; *p* = 0.022) (Table 3). Sports drinks, energy drinks and soft drinks had the highest correlation value (r_s_ = 0.8—0.9). The lowest correlation value (r_s_ = 0.4) was found for caned 100% juice, sweetened juice, milk base juice and sweetened black coffee. The difference between average total daily beverage intake from ABFQ1 and ABFQ2 is 11 ml. However, no significant differences were found between the means (see Table 4).

**Table 3:** Test–retest reliability between ABFQ1 and ABFQ2 among all beverage categories

**Beverage categories**	**Beverage estimated consumption (ml/day)**				**Reliability (test–retest)**	
	**ABFQ1 (*n* = 49)**		**ABFQ2 (*n* = 45)**		**ABFQ1 vs ABFQ2**	
	**Mean ± SD**	**Rang**	**Mean ± SD**	**Rang**	**r**_**s**_	***P*-value**
**Water**	690 ± 569	27–2400	631 ± 476	33–1800	0.5222	≤ .001
**Fresh fruit juice**	15 ± 30	0–200	21 ± 75	0–500	0.6806	≤ .001
**Fresh vegetables juice**	4 ± 26	0–180	6 ± 35	0–235	0.6585	≤ .001
**Caned 100% juice**	7 ± 13	0–60	5 ± 14	0–83	0.4747	≤ .001
**Sweetened juice**	16 ± 73	0–500	11 ± 22	0–83	0.4389	0.002
**Milk juice**	2 ± 9	0–60	2 ± 9	0–60	0.4035	0.006
**Low-fat milk**	46 ± 89	0–400	28 ± 55	0–250	0.7490	≤ .001
**Free fat milk**	2 ± 4	0–20	3 ± 13	0–78	0.7726	≤ .001
**Flavored milk**	2 ± 5	0–20	0 ± 2	0–13	0.2029	0.1812
**Soft drinks**	114 ± 209	0–990	1312 ± 233	0–1065	0.8437	≤ .001
**Iced tea**	24 ± 87	0–550	12 ± 43	0–250	0.7329	≤ .001
**Artificially sweetened soft drinks**	34 ± 66	0–237	46 ± 88	0–355	0.8260	≤ .001
**Artificially sweetened iced tea**	-	-	-	-	-	-
**Unsweetened sugar tea**	52 ± 90	0–325	65 ± 109	0–400	0.7985	≤ .001
**Sweetened tea with/without milk**	103 ± 149	0–650	95 ± 157	0–650	0.8106	≤ .001
**Unsweetened Arabic coffee/Turkish coffee**	96 ± 173	0–1000	92 ± 235	0–1500	0.5923	≤ .001
**Sweetened Arabic coffee/Turkish coffee**	3 ± 16	0–100	5 ± 21	0–100	0.3393	0.022
**Unsweetened black coffee**	149 ± 221	0–946	194 ± 252	0–946	0.6862	≤ .001
**Sweetened black coffee**	35 ± 95	0–472	27 ± 111	0–708	0.4017	0.006
**Flavored coffee**	65 ± 171	0–946	44 ± 88	0–354	0.6001	≤ .001
**Malt drinks**	8 ± 19	0–83	8 ± 19	0–83	0.7828	≤ .001
**Artificially sweetened energy drinks**	6 ± 37	0–250	12 ± 75	0–500	0.5	≤ .001
**Sweetened energy drinks**	14 ± 73	0–500	15 ± 57	0–330	0.8775	≤ .001
**Sport drinks**	4 ± 21	0–133	12 ± 75	0–500	0.9998	≤ .001
**Total beverage**	1504 ± 769	463–4198	1477 ± 750	267–3634	0.7	≤ .001

**Table 4:** Test–retest reliability of total daily beverage intake between ABFQ1 and ABFQ2 (paired t-test)

	**Mean Volume (ml/day)**	**SE**	**SD**	**95% CI**	***P* value**
**ABQ1**	1504	110	785	1284—1725	0.86
**ABQ2**	1477	112	750	1252- 1702	

## Discussion

This study aimed to develop and validate the first online Arabic Beverage Frequency Questionnaire (ABFQ) to assess the total beverage intake. The validity assessment between ABFQ and urine osmolality indicates a negative correlation. However, the correlation was week and not statistically significant (r_s_ = -0.2, *p* = 0.12). In addition, a strong reliability correlation of total beverage between ABFQ1 and ABFQ2 was found (r_s_ = 0.7; *p* < 0.05).

Our validation finding is influenced by the small sample size (*n* = 49) and the variation in participants' characteristics [39]. Also, urine osmolality as a hydration biomarker may be influenced by other factors such as diet, body size, sweat loss and intensive exercises [40–42]. The osmolality of a 24-h urine sample indicates 24-h hydration status and determines the functional surplus of fluids [24, 43]. It can be a quantitative measure to assess beverage questionnaire validity because it negatively correlates with fluid intake in the healthy general population [40–42]. Daily fluid intake directly affects urine osmolality due to antidiuretic hormone influence (ADH). ADH is responsible for fluid reabsorption rate in the kidney; therefore, when fluid intake increases, the antidiuretic level decreases; the kidney reabsorption of fluid decreases; and the urine osmolality increases [40–42].

In this study, the correlation between urine osmolality and average daily intake of total beverage is weak (*r* = 0.2), possibly due to different factors. Primarily, urine osmolality was confirmed to be an excellent indicator of 24-h hydration status [35, 44], and in the validation assessment, we compared 24-h urine osmolality against one day of fluid intake based on the average of 30 days intake recall. A similar correlation (r^2^: 0.20; *p* < 0.001) between urine osmolality, age and average fluid intake was reported by Ferreira-Pêgo *et.all* (2016) [24]. They also reported a bland–Altman parameter estimate of 0.22 between average fluid intake and 24-h urine volume.

Food contains different levels of fluid and moister that contribute to total daily fluid intake depending on the individual and population variation in the type and quantities of foods. Many countries assessed the food contribution to the total fluid intake, such as China(40%), US(19%), Mexico (34.5%), UK(27%) and France (36%) [45]. In Saudi Arabia and Arab countries, there are no data on the food contribution to the total daily fluid intake. Accordingly, assessing the effect of food intake is not possible. Moreover, fruit and vegetables are among the highest food in fluid content (70–95%) [44]. However, Arab countries reported a very low fruit and vegetable consumption [46, 47].

Another factor is having undiagnosed health conditions or undiagnosed kidney dysfunction within participants. However, the urine osmolality range was within the normal range (50–1200 mOsm/kg [48]). Also, participants may not have completed urine samples even though we targeted a well-educated group of participants and collected data on the weekend to avoid any inconvenience at work or university.

Other factors include the general bias of self-reported recall food assessment tools [49]. Besides, the measurement error may result from using an image-based dietary assessment method and providing pictures of available portions size and bottles [50].

Although ABFQ included all beverages consumed by the general population in Arabic countries, it may underestimate the consumption of beverages such as an alcoholic beverages. Alcoholic beverages are prohibited in the religious conviction of Arab countries, and they are either prohibited, restricted or regulated [32, 33].

This study found that for almost all beverage categories and total daily fluid volume, ABFQ was significantly correlated despite the time interval between the ABFQ1 and ABFQ2 (7–20 days). The time interval in our study remained acceptable for repeated frequency measures [39]. Accordingly, ABFQ can be a reliable repeated tool to assess beverage intake and change in consumption patterns over time. Limited variation in the study sample may caused the weak correlation between the two assessments (ABFQ1 and ABFQ2) of flavored milk and sweetened Arabic coffee/Turkish coffee consumption. Sweetened coffee consumption is reported in young adults [51], and in this study, the majority of the study sample (73%) are within the age range of 26–45 years. Flavored milk is also more common in other age groups, such as children. Sweetened iced tea is low in calories (25–40 kcal/100 ml) despite its sugar content (5-6 g/100 ml); therefore, unsweetened iced tea was not reported in both assessments.

Specific Beverage consumption assessment tools were developed for other populations in English and Spanish language. ABFQ is the first tool developed in Arabic and assessed for validity and reliability among the Arabic-speaking population. Nevertheless, a few studies have investigated the consumption of beverages among Arab populations [52–56]. All studies measured the total food consumption by using Food Frequency Questionnaire and one or multiple FIR [52–57]. Only one study used a specific beverage questionnaire [58]. However, the study had some limitations; mainly, the questionnaire was translated from English then the responses collected using paper survey, therefore had limited beverage categories and relied on the individual’s self-estimation of portion size without including the available portion size pictures in the markets.

Developing the ABFQ in an online questionnaire method is one of the main strengths of this study. This is because it allows access to different regional and understudied populations, as well as allows assistance for groups with low health literacy or low education levels groups by providing pictures as guidance of estimating the consumption. Moreover, it being interactive with participants through pictures, videos, and displayed text with or without audio. Besides that, the general advantage of the online questionnaire such as ensuring complete responses, allowing written and visual prompts, allowing immediate and direct responses, allowing accurate scoring and high participant involvement [34]. For example, the recent online beverage Frequency Questionnaire evaluated using pictures to guide the estimation of portion size and container size [36]. One online Arabic questionnaire is used to assess the consumption of soft drinks and the related factor that influence their consumption [55].

Arabic-speaking countries, especially gulf region countries (the United Arab Emirates, Kingdom of Bahrain, Kingdom of Saudi Arabia, Sultanate of Oman, State of Qatar and State of Kuwait [59]), have had a common lifestyle over the years. However, Beverage types, consumption behavior, and drinking utensils vary in these countries. In ABFQ, we sought to enhance the self-recall of portion size and reduce the self-estimation by representing the portion size with pictures and amounts in volume. We showed all local and traditional beverage special cups and utensils found in MENA and gulf population households and markets. For example, Arabic coffee is consumed in most those countries in a special small cup (approximately 50 ml). Another example is the tea which is consumed in some countries in a special small cup (approximately 125 ml), while in others, it is consumed in a regular cup (200 ml).

The study has some limitations; Firstly, we used the biological indicators only to validate the questionnaire, yet, analyzing FIR based on local food composition data was not possible because such data was unavailable. Accordingly, FIR in our study would only increase the burden on participants and may affect the consumption recall causing overestimation or underestimation. While the biological value reports the actual biological status. 24- hour Urine osmolality is found to be the best applicable method to assess hydration status [35]. Other limitations of this study include the convenience and small sample size that allowed for variation between study participants and reduced the statistical power. Also, it caused low consumption reports for some categories that are known for high consumption, such as sports drinks (2 ± 5 ml/day), Sweetened energy drinks (14 ± 73 ml/day), and Free sugar energy drinks (6 ± 37 ml/day). Nevertheless, since this study is a validation study, no specific sample size number is required. Similar published studies reported a sample size ranging from 50 to 160 participants [24, 25, 27, 29, 36, 60].

The future version of this survey may refine beverage groups further based on calories and sugar content from a local database. Milk and Laban has a wide range of products and varies widely from country to country. For example, there is the fresh, long life, flavored, fat reduced or removed and others. It can be gathered in one group or separated according to the target population and local markets. Another possible categorising approach can be based on the sales data to find the most consumed data then assess their consumption. All to achieve better-tailed assessment tools for such broad products.

## Conclusion

The present ABFQ appears to be highly reliable reproducible tool for assessing the intake of different types of beverages among Arabic-speaking consumers. However, the survey could not be validated using 24-h urine osmolality only and therefore other methods including multiple 24 h recalls records should be considered in the future for validation purposes. The Arabic beverage consumption assessment tools is highly valuable for nutrition researchers and clinicians to evaluate habitual patterns and changes in beverage consumption and their influence on health or disease.

## Data Availability

The datasets used and/or analyzed during the current study are available from the corresponding author on reasonable request.

## Abbreviations

ABQ: Arabic Beverage Frequency Questionnaire
mOsm: Milliosmole
ml: Milliliters
r_s_: Spearman’s correlation
MENA: Middle east and north Africa region
NCDs: None communicable diseases
FIR: Food intake records
ABFQ1: First ABFQ
ABFQ2: Second ABFQ
SFDA: Saudi Food and Drug Authority
SMS: Short message service
BMI: Body mass index
WHO: World Health Organization
